# Suppression of HIV-Specific and Allogeneic T Cell Activation by Human Regulatory T Cells Is Dependent on the Strength of Signals

**DOI:** 10.1371/journal.pone.0002952

**Published:** 2008-08-13

**Authors:** Amanda K. Antons, Rui Wang, Spyros A. Kalams, Derya Unutmaz

**Affiliations:** 1 Department of Microbiology and Immunology, Vanderbilt University School of Medicine, Nashville, Tennessee, United States of America; 2 Department of Medicine, Vanderbilt University School of Medicine, Nashville, Tennessee, United States of America; 3 Department of Microbiology, New York University School of Medicine, New York, New York, United States of America; University of California San Francisco, United States of America

## Abstract

Regulatory T cells (Tregs) suppress immune responses against both self and non-self antigens. Tregs require activation through the T cell receptor (TCR) and IL-2 to exert their suppressive functions. However, how strength of TCR signals modulate the potency of Treg-mediated suppression of antigen-specific T cell activation remain unclear. We found that both strength of TCR signals and ratios of Tregs to target cells, either through superantigen, allogeneic antigens or HIV-specific peptides, modified the suppressive ability of Tregs. While human Tregs were able to mediate suppression in the presence of only autologous antigen-presenting cells, this was much less efficient as compared to when Tregs were activated by allogeneic dendritic cells. In another physiologically relevant system, we show that the strength of peptide stimulation, high frequency of responder CD8+ T cells or presence of high IL-2 can override the suppression of HIV-specific CD8+ T cells by Tregs. These findings suggest that ratios and TCR activation of human Tregs, are important parameters to overcome robust immune responses to pathogens or allogeneic antigens. Modulating the strength of T cell signals and selective enhancement or depletion of antigen-specific Tregs thus may have implications for designing potent vaccines and regulating immune responses during allogeneic transplantation and chronic infections.

## Introduction

The role of regulatory T cells (Tregs) in maintaining control of immune responses in the context of autoimmunity, transplantation and infectious diseases is now well-established. Tregs are CD4^+^ T cells which express the transcription factor FoxP3. Phenotypically, human Tregs constitutively express high levels of CD25, and the memory marker CD45RO, various activation markers [1] and lack the expression of IL-7 receptor, CD127 [2], [3]. Tregs are hyporesponsive to TCR stimulation, as displayed by their impaired proliferative capacity and cytokine secretion upon stimulation in vitro [1], [4]. Tregs require activation through the T cell receptor (TCR) [5] and cell-to-cell contact for suppression [6], [7]. Loss of Tregs or lack of Treg function in vivo results in severe autoimmune disorders in humans and mouse models [8], [9], [10].

A favored model of Treg differentiation proposes that a population of thymocytes that recognize self-MHC/antigen complexes, escape negative selection and enter the FoxP3^+^ Treg differentiation pathway [11], [12], [13], [14]. However, a recent study that characterized hundreds of TCRs expressed by FoxP3- naïve CD4^+^ T cells, or Tregs showed that non-self-antigens are the cognate specificities of FoxP3+ Tregs [15]. In support of the notion that Tregs recognize antigens other than self, several reports showed that Tregs recognize exogenous microbial antigens derived from viral [16], [17], [18], [19], [20], bacterial, fungal [21], [22], [23], [24], [25] and parasitic origins [25], [26], [27], as well as allogeneic tissues during transplantation [28], [29]. It has been unclear whether these Treg responses are antigen-specific or rely on inherent cross reactivity, or are bystander activations with self-antigen.

Conflicting results in numerous model systems in identifying the specificity of the antigen during suppression have made it difficult to identify precise parameters and mechanisms of Treg-mediated suppression [15]. Transwell experiments have shown that direct cell contact between Tregs and target cells is required [6], [7], or that they secrete inhibitory soluble mediators that act at very close proximity of target cells. IL-2 is also involved in the development and function of Tregs [30], [31], [32], [33]. IL-2 signaling appears to be required for both the thymic development and peripheral expansion/maintenance of Tregs [31], [32], [33]. How IL-2 is involved in T cell function remains controversial. Tregs from mice containing a FoxP3 knock-in-allele engineered to lack IL-2 or the IL-2α chain were both capable of suppressing T cell proliferation in vitro [31]. In contrast, it has been shown that suppression is completely abrogated by selective blocking of the IL-2 receptor on the Tregs [30].

Understanding the mechanisms of Treg-mediated suppression could be important in controlling a variety of disease processes. For example by enhancing Tregs during transplantation, it may be possible to suppress Graft-versus-host-disease. Tregs may also be important in controlling the immune response during chronic infections [1], [34], [35] or conversely unwanted or aberrant Treg function may result in inefficient responses to infectious diseases [35], [36]. Of particular interest is HIV infection, which has the hallmark of chronic immune activation [37], [38]. We and others have shown that Tregs are targeted by HIV and are depleted during late stages of the disease, potentially contributing to hyper-activation [1], [36]. It has also been shown that Tregs can blunt immune responses against HIV infection in vitro [17], [39] as well as other infectious diseases [21], [25], [40]. However, it is not yet clear how efficiently Tregs can suppress HIV-specific immune responses in vivo or whether they can directly recognize HIV antigens. Therefore, understanding the mechanisms of Tregs during HIV and other chronic viral infections [41], is critical to develop better preventive and therapeutic vaccine approaches.

We sought to better understand the relationship between the strength of TCR signals and ability of Tregs to suppress immune activation in three different in vitro antigen-specific models: 1) Superantigen, 2) Allogeneic and 3) HIV-specific peptides. We show that the efficiency of human Treg-mediated suppression is strongly dependent on the magnitude of antigen-specific signals directed towards both the target and Tregs in all three in vitro models. We also found that increasing the ratios of Tregs to target cells, partly overcomes stronger TCR signals. These findings underscore the intricate balance between Tregs and effector cells in generating, maintaining and suppressing immune responses.

## Results

### Strength of TCR activation and Treg numbers modulate the level of Treg mediated suppression

**Figure 1:**
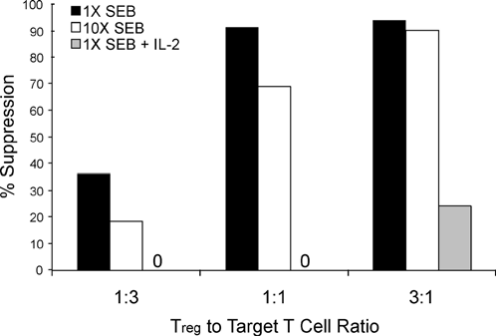
Strength of TCR activation and Treg numbers modulates level of suppression. CD4+ naïve T cells were isolated and CFSE labeled. Unlabeled control T_M_ or Tregs were added to the cultures at the target to Treg ratio as indicated. Cells were stimulated with 2 different concentrations of SEB, or in the presence of IL-2. Shown is percent suppression by the Tregs when normalized to T_M_ or T_N_ control. The % suppression on Y-axis is calculated as follows: We first determined the percent of CFSE labeled target cells dividing in response to the stimuli alone. We then determined the proliferative percentage of cells in the presence of Tregs or control effector T cells. The percent suppression was then calculated by percent reduction in proliferation of the target cells in the presence of effectors as compared to target cells alone. As an example, in one experiment 40% of the CFSE labeled T cells divided in response to SEB stimulation. In the presence of Tregs the same cells divided only 4%. Thus, we represent this as 90% suppression. Data shown are representative of three replicate experiments.

In in vitro assays, ratios of Tregs to target cells that are between 1∶1 to 1∶4 are typically used for suppression of T cell activation [1], [6], [7], [42]. Treg mediated suppression is also dependent on the strength of TCR activation and is more potent at suboptimal levels of activation [6], [7]. We further investigated how the the strength of TCR signals and Treg ratios affected suppression of T cell activation. Tregs and naïve CD4^+^ T cells (T_N_) were purified from healthy human peripheral blood. Target CD4^+^ T cells were labeled with CFSE. These cells were then activated with dendritic cells (DCs) pulsed with different concentrations of a superantigen (SEB), either in the presence of non-labeled control memory CD4+ T cells (T_M_) or Tregs. As expected, a 1∶1 ratio of Treg to target cell yielded efficient suppression of T cell activation (Figure 1). Increasing the concentration of SEB by 10-fold reduced the efficiency of Treg-mediated suppression at 1∶1 ratio of Tregs to target cells (Figure 1). However, when Tregs to Target ratio was increased to 3∶1 ratio Tregs were also capable of suppressing 10-fold higher SEB concentrations (Figure 1). Whereas, addition of IL-2 to the cultures greatly reduced the effectiveness of Tregs to suppress T cell activation even at high ratio of Treg to target cells (Figure 1).

### Suppression of allogeneic and auto-antigen mediated T cell activation by Tregs

**Figure 2:**
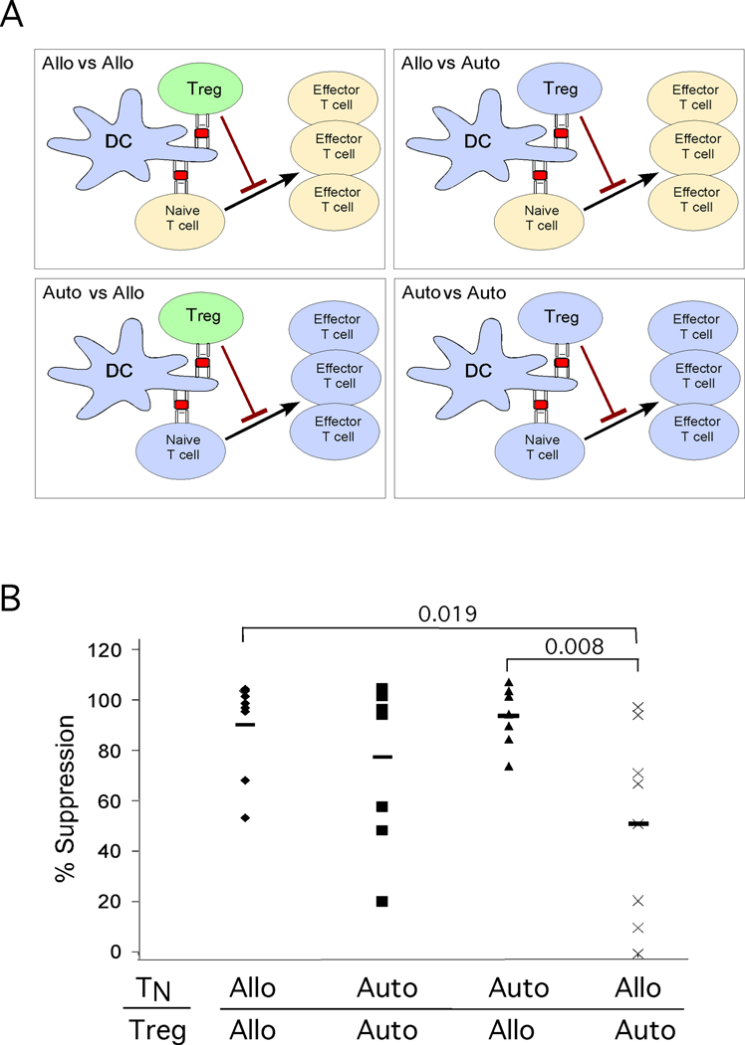
Treg mediated suppression of allogeneic T cell responses. (A) Experimental setup allogeneic vs. autologous stimulation of CFSE labeled target cells (T_N_), and Tregs. (B) Conditions were designed as depicted in Figure 2A. Shown is percent suppression by the Tregs normalized to control T cells. Each point represents an individual experiment. in total 8 experiments separate experiments were performed. P values are shown above the brackets as calculated by Student's t Test.

Tregs could play an important role in controlling graft-versus-host-disease (GVHD) during allogeneic transplantations. It is not clear whether recognition of allo-antigens by Tregs render them more potent in their suppressive capacity as compared to Tregs that recognize autologous antigens. Based on our previous findings we hypothesized that suppression of allo-specific immune responses would be more efficiently mediated by Tregs activated by allogeneic compared to autologous DCs. To test this we devised an in vitro suppression assay, whereby we purified both T_N_ and Tregs from the peripheral blood of 8 different human subjects. We also generated DCs from each of these donors as described [43]. We next set up matched experiments to mix with CFSE labeled T_N_ cells with Tregs and DCs in either of these conditions: 1) Allogeneic vs. allogeneic: both Tregs and T_N_ cells were from the same donor and stimulated with allogeneic DCs from another donor. 2) Autologous vs. autologous: both Tregs and T_N_ cells were from the same donor stimulated with autologous DCs. 3) Autologous vs. allogeneic: T_N_ cells from one donor were stimulated with the same donor autologous DCs, in the presence of Tregs from a different donor therefore recognizing DCs as allogeneic. 4) Allogeneic vs. autologous: T_N_ cells from a donor were stimulated with DCs from a different donor, in presence of Tregs that were isolated from the same donor as the DCs (Figure 2A).

We observed that allogeneic Tregs were very potent in suppressing activation of both autologous and allo-specific CD4^+^ T cell proliferation (Figure 2B). Autologous Tregs were also relatively efficient in suppressing CD4+ T cell proliferation in response to presumably self-antigens presented by autologous DCs (Figure 2B). However, when both DCs and Tregs were from the same donor, these Tregs were much less efficient in suppressing allogeneic CD4+ T cell activation (Figure 2B). In summary these results show that while Tregs that potentially recognize self-antigens are capable of suppressing self-antigen mediated T cell proliferation, they are less effective when responder T cells are activated by allogeneic stimuli. In contrast, when Tregs recognize allo-antigens they become more potent suppressors. Taken together these findings suggest that self-reactive Tregs are not as potently activated as allogeneic antigens and that this strength of signal is a critical parameter in efficient Treg-mediated suppression.

### Suppression of HIV-specific CD8 T cell activation by auto- or allogeneic Tregs

We next sought to recapitulate the previous findings in a physiological immune response model, where a defined antigen-specific activation can be precisely controlled. We utilized a cohort of HIV+ individuals who are considered HIV+ slow-progressors as defined by viral loads of less than 50,000 copies/ml in the absence of antiviral therapy that have been well characterized in respect to their HIV-specific immune responses. The viral and immunological profiles of the infected individuals are shown in Table 1.

**Table 1:** Immunological characteristics of HIV subjects.

Patient #	Infection (years)	CD4 Count	CD8 Count	Viral Load	%FoxP3+CD4+	% KF11+ CD8
1	16	661	409	215	1.22	2.9
2	11	950	437	5917	1.81	10.1
3	15	743	414	50	1.27	1.98
4	8	700	1500	21339	0.77	9.85
5	3	1353	858	3288	1.44	9.78

**Figure 3:**
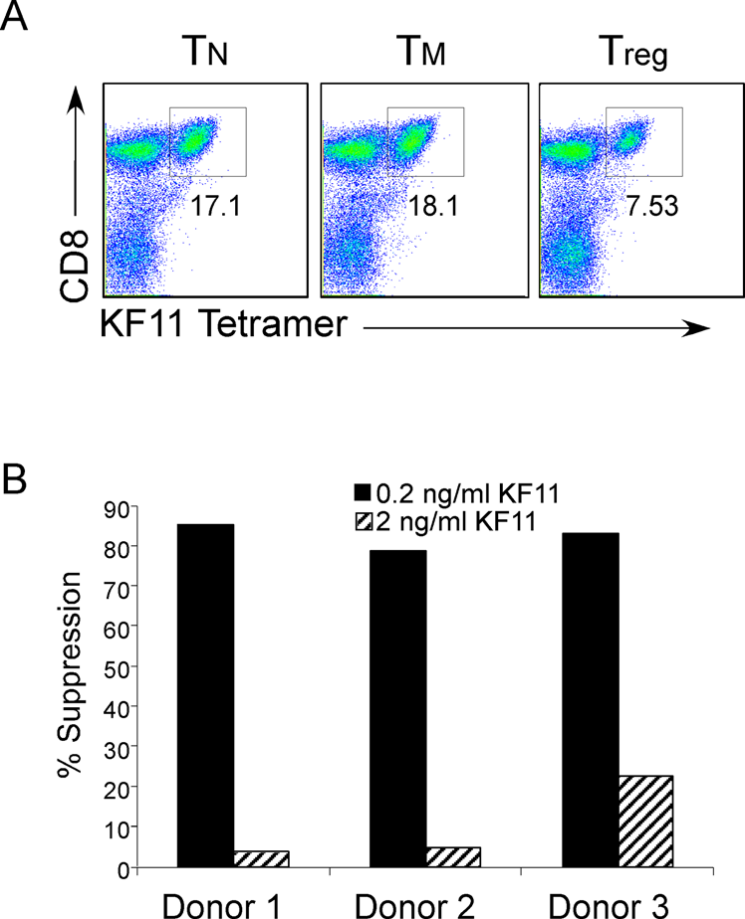
Allogeneic Tregs are capable of suppressing antigen specific proliferation. (A) To identify HIV-peptide specific CD8+ T cells PBMC cultures were stained with antibodies for CD3, CD4, CD8, and tetramer specific for HIV peptide, KF11. Plots shown are gated on CD3+ T cells, and tetramer positive cells were also CD8+. (B) Tregs or control T cells were isolated from HIV-negative individuals. PBMC from HIV+ individuals were CFSE labeled and mixed with either HIV-negative allogeneic T or Tregs, and stimulated with 0.2 or 2 ng/ml KF11 HIV peptide. Cells were stained with KF11 Tetramer, and level of proliferation was assessed d5 post activation. Shown is percent suppression by Tregs as described in Figure 1. Data is representative of 5 separate experiments.

**Figure 4:**
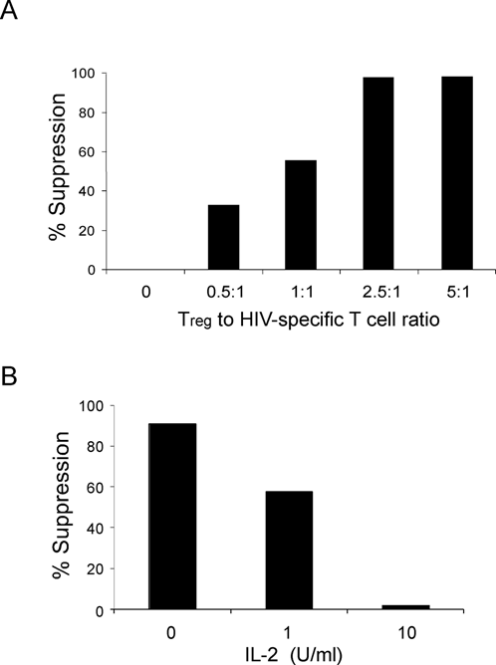
Cell ratios and IL-2 effect Treg suppression of HIV specific proliferation. (A) Tregs or control T_N_ cells were isolated from HIV-negative individuals. PBMC from HIV+ were CFSE labeled and stimulated as indicated in Figure 3B. Tregs or control T cells were added at the ratio indicated. Shown is percent suppression by Tregs as described (Figure 1 legend). (B) HIV-positive PBMC was stimulated with KF11 peptide for 5 days in the presence of allogeneic Tregs and increasing concentrations of IL-2. Data is representative of three separate experiments.

PBMC from HIV+ individuals were labeled with CFSE and stimulated with HIV peptide (HLA-B57 restricted), KAFSPEVIPMF (KF11) in the presence or absence of Tregs or CD4+ T cell subsets from an HIV negative subject. We then assessed the proliferation of the KF11-specific CD8 responses, after 5 days, by co-staining with HLA-class I tetramers pulsed with KF11 peptides, and CD8, CD4, and CD3 antibodies (Figure 3A). Allogeneic Tregs were very efficient in suppressing the proliferation of CD8+ HIV specific immune responses at suboptimal peptide concentrations (Figure 3B). However, suppression was much less efficient when the concentration of KF11 peptide was increased 10-fold (Figure 3B). At lower ratios of Tregs to HIV specific CD8^+^ cells the Tregs could not fully suppress proliferation of KF11-specific T cells (Figure 4A). Addition of IL-2 to PBMCs stimulated with HIV specific peptide also affected the ability of allogeneic Tregs to suppress the proliferation of a HIV specific response (Figure 4B), in agreement with our previous result (Figure 1).

**Figure 5:**
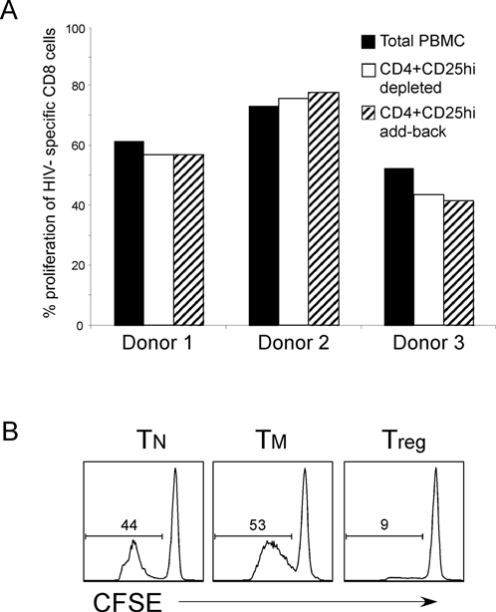
Depletion of Tregs does not result in increased KF11-specific proliferation. (A) PBMC from HIV+ individuals were stained with antibodies directed against CD4 and CD25. CD4+CD25^hi^ cells were removed from the PBMC by sorting CD4+CD25^hi^ T cells using FACS. Total or CD4^+^CD25^hi^ depleted, or PBMC were then CFSE labeled and stimulated with KF11 peptide. Cells were stained as in Figure 3A, and percentage of KF11-specific CD8^+^ T cells that proliferated is shown for 3 representative donors. In conditions that CD4^+^CD25^hi^ cells were added back to cultures, they were added at a ratio of at least 3∶1 CD4^+^CD25^hi^ to labeled PBMC. (B) Treg, T_N_ or T_M_ cells were isolated from purified CD4^+^ T cells from HIV+ individual, based on expression of CD45RO and CD25. T_N_ cells from the same donor were CFSE labeled, and unlabeled T_N_, T_M_ or Tregs were added at a ratio of 1∶1 target to effector T cells. Cultures were stimulated with SEB pulsed DCs, and fixed on day 4–5 post activation. Histogram of CFSE labeled target cells are displayed, and the percentage of cells that have proliferated as shown in gates. Representative is one out of three experiments.

To determine whether autologous Tregs from PBMCs of HIV controllers can also have suppressive function against HIV-specific CD8^+^ T cells, we depleted CD25^+^ Tregs from the PBMC of these individuals and tested their responses to peptide stimulation. Similar to above experiments CD25^+^ T cell depleted PBMC were labeled with CFSE and HIV-specific CD8+ cells were identified by directed staining with KF11-tetramer, and proliferation was assessed 5 days post-stimulation. We did not observe any significant enhancement of CD8^+^ T cell proliferation in any of the donors tested with wide range of peptide concentrations when Tregs were depleted from PBMCs (Figure 5A). Similar results were obtained when CD8+ T cells were stimulated with Gag peptide pools (data not shown). In addition, when we added back the sorted autologous CD25^+^ cells into these activation cultures at high ratios compared to responder CD8+ T cells (5∶1) there was still no suppression of HIV-specific proliferation (Figure 5A).

One possibility for inability of autologous Tregs isolated from HIV-infected donors could be that these cells have lost their suppressive capacity. In order to rule out this possibility we tested their capability to suppress CD4^+^ T cell activation in a superantigen-mediated activation system described in Figure 1. We found that Tregs isolated from HIV+ donors were equally functional in their suppressive ability (Figure 5B). Together, these findings suggest that in these HIV-infected subjects, activation of Tregs by presumptive self-antigens only is not sufficient to suppress HIV-specific CD8+ T cell activation.

## Discussion

In this study, we found that the activation signals to the target T cells and Tregs can modulate the suppressive function of the Tregs. Increasing the strength of signal or presence of IL-2 can override the Treg-mediated suppression, which we show in the context of three different in vitro models of antigen-specific T cell activation. In contrast, higher frequency of Tregs and stronger stimuli through their TCR results in better suppression of effector T cell activation, The data presented here provides us with better insight into the delicate balance between activation signals and suppressing an antigen specific response.

We showed that the strength of TCR and IL-2 signals affects the magnitude of suppression that can be achieved by the Tregs. However, it remains to be determined whether direct suppression of initial activation of antigen-specific T cells by Tregs extinguishes the immune response at a population level of T cells, without directly inhibiting every responder T cell. This could potentially happen in a scenario where Tregs stop few potent responder T cells from secretion of cytokines, thus limiting the availability of growth factors such as IL-2 to neighboring cells that may require IL-2 for efficient activation. Indeed, addition of IL-2 to in vitro activation cultures overrides Treg-mediated suppression of target T cells during both SEB and allogeneic stimulation. It is also possible that IL-2 changes the activation threshold needed for Treg suppressive capability at the cell-to-cell interaction level. While not mutually exclusive, these data highlight the important double-edged role of IL-2 both in enabling Tregs to suppress and target cells to escape Treg-suppression.

When we stimulated Tregs only in the presence of autologous APCs the suppression observed was less potent compared to suppression by Tregs stimulated with SEB, allogeneic DCs or HIV-peptide. These findings would predict that during the course of an immune response, Tregs that recognize antigens from pathogens develop in parallel to conventional effector/memory T cells. In support of this prediction, we recently provided evidence that Tregs can develop from a naïve T cell precursor when activated in vitro through their TCR [44]. Our findings do not rule out the possibility that autologous or bystander Treg activation play a role in the suppression of HIV specific immune responses. However, we speculate that suppression of HIV-specific activation by Tregs recognizing self-antigens would not be efficient unless HIV-specific CD8 cells are at very low frequencies.

During HIV infection, global control of T cell activation by Tregs could conceivably limit cellular targets of HIV, as T cell activation is required for a successful infection [16]. In contrast, it has been suggested that HIV-immune dysfunction is associated with suppression of HIV-specific effectors by Tregs, leading to an inefficient immune response against HIV [36], [39], [45], [46]. Several reports have shown that depletion of Tregs can amplify CD8^+^ T cell responses [17], [46]. One plausible reason why in our experimental system we did not observe similar results could be that the patient population we used has very potent and high frequencies of CD8^+^ T cell responses, thus as we showed, rendering them more difficult to suppress. It is also possible that the affinity of CD8+ T cells for this HIV peptide is too strong for autologous Tregs to overcome. Indeed, increasing the dose of peptide concentration by 10-fold was able to overcome the suppression mediated even by the allogeneic Tregs. Further, in our system, it is also highly unlikely that Tregs are activated by CD8+ specific HIV-peptide, therefore they would have to rely on self-antigens in order to be activated and display their suppressive function. In other experimental systems, where T cells are activated by complete HIV antigens or viral particles, Tregs could also potentially recognize HIV antigens and thus conceivably more potent in suppressing HIV-specific activation.

Defining the determinants of Treg-mediated regulation of allogeneic immune responses is essential for therapeutic application of Tregs in transplantation tolerance and control of GVHD [28], [29], [47], [48]. We have confirmed that allogeneic stimulation of Tregs renders them capable of suppressing the activation and proliferation of conventional T cells. In order to successfully prevent GVHD after an allograft-transplant, donor Tregs that are transferred or develop in the host should be able to suppress most of allo-specific CD4+ and CD8+ T cells [28]. However, our data suggest that Tregs do not necessarily have to recognize the same allo-antigens as the effector T cells. Successful inhibition of allo-specific T cell activation would only require that some Tregs are also activated at the same time, even if through different antigens. Based on these postulates, a potential treatment approach would be to isolate Tregs from donor blood prior to the transplantation, which would then be activated and expanded with allogeneic DCs from the host recipient. These Tregs can then be infused to patients with the transplant. Because Tregs have limited in vitro expansion potential [49], [50], recently identified naïve Tregs, which have greater proliferative capacity could be useful for this approach [44], [51], [52], [53].

In summary, our results indicate that the strength of TCR signals and IL-2 can override Treg-mediated suppression when Treg numbers and activation are limiting. In the context of HIV, both controlling immune activation and generation of effective HIV-specific immune responses may need to be balanced. Thus, Treg responses during HIV infection may require fine-tuning depending on the stage of the disease. Approaches to modulate this delicate balance will be important when designing vaccines for HIV or other infectious diseases and controlling the immune response during transplantation or in chronic immune activation.

## Materials and Methods

### HIV donors

HIV-positive subjects were recruited through the Comprehensive Care Center (Nashville, TN) and all subjects were HLA class I typed (4 digit resolution) (DCI, Nashville, TN). All subjects were antiretroviral naïve at the time of study with a range of CD4+ T cell numbers from 144 to 1260/mm^3^ and log viral load measurements from 1.7 to 4.25 copies/ml. This study was approved by the Vanderbilt University Medical Institutional Review Board and all subjects provided written informed consent.

### Cell Isolation and culture

The blood obtained from healthy donors for this study have been reviewed and approved by Vanderbilt and NYU School of Medicine IRB committees. PBMC were isolated from blood of donors through Ficoll-Hypaque (Pharmacia). Resting CD4^+^ T cells were purified using CD4^+^ dynabeads (Dynal) as previously described [54] and were at least 99.5% pure as determined by post-purification FACS analysis. To purify T_N_, T_M_, Treg and T_Nreg_ subsets, purified CD4^+^ cells were stained with CD25 and CD45RO antibodies and CD45RO^−^CD25^−^ (T_N_) and CD45RO^+^CD25^hi^ (Treg) were sorted on a FACS Aria^TM^ flow cytometer. The culture media used in all experiments was RPMI (Life Technologies) and was prepared as described [54]. All cytokines were purchased from R&D Systems. Monocyte-derived dendritic cells (DCs) were generated as described [54].

### FACS Analysis

T cells were stained with the relevant antibody on ice for 30 min in PBS buffer containing 2% FCS and 0.1% sodium azide. Cells were then washed twice, fixed with 1% paraformaldehyde, and analyzed with a FACSCalibur® or FACSAria® flow cytometer. Live cells were gated based on forward and side scatter properties and analysis was performed using FlowJo software (Tree Star). Antibodies used in experiments were: CD4-APC Cy7, CD8-PB, CD3-PE Cy7, CD14-PerCP, CD19-PerCP, CD56-PE Cy5.5 (All from BD Pharmigen) and B57-KF11 Tetramer-PE (Beckman Coulter).

### T cell suppression Assay

Purified CD4^+^ T cells were FACS sorted, as described above, based on expression of CD25 and CD45RO. Target PBMC (peptide stimulated suppressions) T_N_ or T_M_ (SEB stimulated suppression) cells were CFSE labeled. Purified cells were first washed and resuspended in (PBS). While vortexing the cells, CFSE was added at a final concentration of 5 µM. The mixture was vortexed for an additional 15 s and incubated at 37°C for 3 min. Labeling was quenched by addition of 50% fetal calf serum in PBS. Cells were washed once more with 50% serum PBS, followed by two washes with RPMI-supplemented medium. CFSE-labeled target T cells and autologous or allogeneic DCs were cultured in a round-bottom well of a 96 well plate, in the presence of HIV peptide KF11 (2-0.02 ng/ml), or SEB (10-0.001 ng/ml), or no stimulant. Cells were collected, stained, and fixed with 1% paraformaldehyde, and analyzed on day five post-activation.
